# Oral *Lactobacillus* species and their probiotic capabilities in patients with periodontitis and periodontally healthy individuals

**DOI:** 10.1002/cre2.740

**Published:** 2023-04-20

**Authors:** Arghavan Etebarian, Tahere Sheshpari, Kourosh Kabir, Hanieh Sadeghi, Abouzar Moradi, Avin Hafedi

**Affiliations:** ^1^ Oral and Maxillofacial Pathology Department, School of Dentistry Alborz University of Medical Sciences Karaj Iran; ^2^ Microbiology Department, School of Medicine Alborz University of Medical Sciences Karaj Iran; ^3^ Community Medicine Department, Dietary Supplements and Probiotic Research Center Alborz University of Medical Sciences Karaj Iran; ^4^ Student Research Committee Alborz University of Medical Sciences Karaj Iran; ^5^ Periodontology Department, School of Dentistry Alborz University of Medical Sciences Karaj Iran

**Keywords:** lactobacillus, oral cavity, periodontitis, probiotics

## Abstract

**Objectives:**

This study aimed to identify oral *Lactobacillus* species and characterize their adhesion properties and antibacterial activity in patients with periodontitis compared with periodontally healthy individuals.

**Materials and Methods:**

Three hundred and fifty‐four isolates from the saliva, subgingival, and tongue plaque of 59 periodontitis patients and 59 healthy individuals were analyzed. Oral *Lactobacillus* species were identified through the culture method in the modified MRS medium and confirmed by molecular testing. Moreover, the radial diffusion assay and cell culture methods were used to determine the antibacterial activities of oral strains against oral pathogens and their adhesion activity in vitro.

**Results:**

67.7% of the cases and 75.7% of the control samples were positive for the *Lactobacillus* species. *Lacticaseibacillus paracasei* and *Limosilactobacillus fermentum* were the dominant species in the case group, whereas *Lacticaseibacillus casei* and *Lactiplantibacillus plantarum* were dominant in the control group. *Lactobacillus crispatus* and *Lactobacillus gasseri* had higher antibacterial effects against oral pathogens. Moreover, *Ligilactobacillus salivarius* and *L. fermentum* demonstrated the highest ability to adhere to oral mucosal cells and salivary‐coated hydroxyapatite.

**Conclusion:**

*L. crispatus, L. gasseri, L. fermentum*, and *L. salivarius* can be introduced as probiotic candidates since they demonstrated appropriate adherence to oral mucosal cells and salivary‐coated hydroxyapatite and also antibacterial activities. However, further studies should be conducted to assess the safety of probiotic interventions using these strains in patients with periodontal disease.

## 1 INTRODUCTION

Dental caries and periodontitis are infectious diseases associated with dysbiosis of the microorganisms in dental plaque biofilm. *Porphyromonas gingivalis* is the key factor in periodontal disease development (Hirasawa & Kurita‐Ochia, 2020). Periodontitis can have a negative effect on quality of life (Ferreira et al., 2017) and is associated with systemic disorders such as cardiovascular disease (Sanz et al., 2020), metabolic syndromes (Pirih et al., 2021), and respiratory infections (Kelly et al., 2021). Changes to the population of indigenous bacteria balance in the oral cavity are associated with the development of periodontitis, with an increase in the population of pathogenic bacteria and a decrease in the population of beneficial bacteria (Curtis et al., 2020). New bacterial treatments can be used as alternative treatments for infections caused by pathogens, which regulate the oral microbiota and remove pathogenic bacteria (Bosch et al., 2012).

Probiotics have demonstrated promising results as anti‐inflammatory, anticancer, antimicrobial, antioxidant, and immunomodulating agents (Lu et al., 2021; Sankarapandian et al., 2022) and have been considered to balance the natural microbiome in the human body, such as the urogenital tract, the respiratory system, skin, and the oral cavity (Shimauchi et al., 2008). In addition, they can effectively prevent and treat several infectious diseases in the oral cavity, including periodontitis, tooth decay, and halitosis (Bustamante et al., 2020; Shimauchi et al., 2008; Teughels et al., 2013). Bifidobacteria and lactic acid bacteria (LAB) are the most common probiotic strains, such as *Lactobacillus*, which is the most crucial group of probiotics that produces lactic acid in the gastrointestinal system (Shokryazdan et al., 2014). As probiotics, Lactobacilli have antibacterial activities and interfere with the growth of surrounding microbiota. In addition, they produce organic acids such as lactic acid and acetic acid, leading to a low pH (Cuozzo et al., 2000; Hirasawa & Kurita‐Ochia, 2020). *Lactobacillus* species can reside in various parts of the oral cavity, such as oral mucosa, hard tissue, saliva, tongue, and supra‐ and subgingival plaques (Terai et al., 2015). One of the most critical characteristics of lactobacilli is its ability to adhere to epithelial cells and produce antibacterial substances (Na et al., 2020; Terai et al., 2015). The duration of transfer of foods into the oral cavity is shorter than that in other areas of the gastrointestinal system. The oral bacteria are transferred into the stomach together with the saliva. Therefore, oral probiotics must be capable of adhering to the oral tissues. In addition, the antibacterial activities hinder the growth of pathogenic bacteria through antibacterial substances in the microbial supernatant (Kolenbrander et al., 2002). With the ever‐increasing use of these bacteria in treating oral diseases, it is essential to determine the properties of oral probiotics to select the appropriate strain and optimize the results of bacterial treatments (Terai et al., 2015). More knowledge about oral lactobacilli could help understand oral dysbiosis and might provide measures for novel therapeutic agents. There is considerable evidence on lactobacilli and their probiotic potential (Abdel‐Daim et al., 2012; Bosch et al., 2012; Hirasawa & Kurita‐Ochia, 2020). With the current interest in probiotics (Raghuwanshi et al., 2015), this is the first study to identify and characterize oral *Lactobacillus* species from the samples collected from periodontitis patients and periodontally healthy individuals in an Iranian population. Moreover, this study aimed to investigate the *Lactobacillus* adhesion activity to oral mucosal cells and salivary‐coated hydroxyapatite (S–HA), besides the antibacterial activity of these strains against oral pathogens.

## 2 MATERIALS AND METHODS

### 2.1 Study subjects

Patients seeking periodontal or dental care in the Alborz Dental School were screened, and 118 volunteers were assigned to two groups (with similar age and gender distribution, age range 25–70 years). (1) Case group: patients with periodontitis classified as moderate to severe (Caton et al., 2018), with at least four sites with probing pocket depth (PPD) > 3 mm, clinical attachment loss, bleeding on probing, bone loss, and (2) Control group: healthy subjects with at least 24 natural teeth (excluding third molars), probing depth (PD) ≤ 3 mm, and without oral predisposing factors causing local irritation or plaque retention (Gomes‐Filho et al., 2018; Kuru et al., 2017). Before clinical examinations, medical and dental history was obtained.

Exclusion criteria were as follows: history of smoking, history of diabetes, pregnancy, breastfeeding, autoimmune disease, necrotizing periodontal disease, history of periodontal treatment in the past 6 months, been receiving antibiotics or anti‐inflammatory drugs in the past 4 months, and patients indicated for prophylactic antibiotics before routine dental treatments.

An informed consent, including the aim and content of the survey, was signed by all the study subjects. The study protocol was approved by the Alborz University of Medical Sciences Ethics Committee and was conducted following the Helsinki Declaration of 1975 (revised 2013).

### 2.2 Collection of oral specimens and isolation of *Lactobacillus* from sample cultures

Oral specimens were collected from subgingival plaque, tongue, and saliva. The teeth were isolated using cotton rolls and dried with compressed air to avoid contamination with saliva. Subgingival plaque samples were pooled from the posterior first molars in each quadrant or, in case of the plaque absence on these teeth, from a tooth with the deepest PPD and significant amounts of dental plaque were obtained using sterile Gracey curettes (Teughels et al., 2013). Microbial tongue samples were collected using a sterile cotton swab rotated five times on an area of 2.16 cm^2^ on the left side of the tongue dorsum (Teughels et al., 2013). Unstimulated saliva samples were collected according to a protocol described by Navazesh (1993). Participants were asked to allow saliva to accumulate on the floor of the mouth for 1–2 min, following which they spat 2–3 mL of saliva into a specimen tube (Navazesh, 1993).

The saliva, subgingival, and tongue plaque samples were collected and diluted in *Lactobacillus* selective (LBS) broth as a transport medium. Then, they were cultured on modified DeMan, Rogosa, and Sharpe (MRS) agar plates (Difco‐Merck) and the LBS agar medium for additional confirmation (Vancomycin‐HCL, Bromocresol green, and Cysteine Hydrochloride were added to MRS to specifically identify lactobacilli strains). The plates were incubated anaerobically at 37°C for 3 days. Afterward, catalase and oxidase tests were performed, and wet mounts and Gram‐stained slides were prepared and examined under a microscope (Olympus Corporation) to ensure the existence of gram‐positive, bacillus‐shaped, and catalase‐ and oxidase‐negative bacteria. Isolated colonies with typical characteristics of lactobacilli were picked from the plates and stored at −80°C in MRS broth containing 20% Glycerol (Patel, 2016). The 24‐h microbial suspension pH of the lactobacilli strains ranged between 3.2 and 5.4.

### 2.3 DNA extraction using the modified salting‐out method

The modified salting‐out method was used for the extraction of bacterial genomes. First, 100 μL of bacterial cells were resuspended in 250 μL of lysis buffer (1 M Tris‐HCl pH = 8, 0.5 M EDTA pH = 8, and 5 M NaCl pH = 8) by vortexing; 100 μL of sodium dodecyl sulfate (SDS) 10%w/v, and 3 μL of proteinase K (Sigma‐Aldrich) were added and vortexed gently. It was incubated at 37°C for 24 h. Then, 6 M NaCl was added and centrifuged at 3400 rpm at 10°C for 30 min. The supernatant was transferred to new microtubes, and cold ethanol was added. Next, the tubes were centrifuged at 4000 rpm at 10°C for 17 min. The supernatant was discarded, and 500–1000 μL of 70% ethanol was added to the tube. Then, the tubes were centrifuged at 12,000 rpm at 10°C for 5 min (repeated twice). Finally, the tubes were air‐dried, and 100 μL of elution buffer was added. The tubes were kept at 4–5°C for 2–3 days to dissolve DNA completely. The quality of the extracted DNA was confirmed using electrophoresis‎ on agarose gel 0.8% (wt/vol) and visualized under UV light. *Lactobacillus acidophilus* and *Lactiplantibacillus plantarum* were extracted using the DNA extraction kit and used as positive controls (Chacon‐Cortes et al., 2012).

### 2.4 Identification of isolates based on 16S rDNA genes polymerase chain reaction–restriction‐fragment‐length polymorphism and Sanger sequencing

The isolates were identified at the species level using restriction fragment length polymorphism analysis of polymerase chain reaction‐amplified 16S ribosomal DNA genes (16S rDNA polymerase chain reaction–restriction‐fragment‐length polymorphism) and Sanger sequencing. Therefore, the genomic DNA samples were amplified by polymerase chain reaction using the universal primers: 27F (5′ AGAGTTTGATCMTGGCTCAG 3′) and 1525R (5′ AAGGAGGTGWTCCARCC 3′) (SinaClon) for the 16S rRNA gene.

The polymerase chain reaction was carried out in a thermocycler (Eppendorf). Thirty‐two cycles of amplification were carried out in a final volume of 25 μL, including 5 μL of DNA template and amplification mixture, which contained 0.25 μL of each primer, 0.3 μL of dNTPs, 2.5 μL of 10× amplification buffer, 0.5 μL of MgCl_2_, and 0.2 μL of Taq DNA polymerase. The polymerase chain reaction amplification program consisted of an initial heating step at 95°C for 5 min, 30 cycles at 95°C for 45 s, 60°C for 1 min, 72°C for 15 min, and a final extension step at 72°C for 12 min. At the end of the incubation, the amplification products were separated by electrophoresis through 1% (w/v) agarose gel in 1× TBE buffer and visualized under UV illumination. A 100–3000 bp ladder (SinaClon) was used to estimate the fragment size of the amplicons generated. The bp1545 bands indicated that the lactobacilli DNA product was obtained (Nikolic et al., 2008).

The polymerase chain reaction products were digested using the restriction endonucleases Taq I and Hae III (Thermo Fisher Scientific). The products of enzymatic reactions were analyzed by electrophoresis in 1.5% (wt/vol) agarose gels. The isolates were categorized into 10 groups based on the weight and the number of bands obtained; then, 53 representative strains (3–4 isolates from each group with different restriction‐fragment‐length polymorphism patterns) were selected, and Sanger sequencing was conducted by the same primers used for polymerase chain reaction on ABI 3500 automated sequencers (Applied Biosystems). The identified sequences were analyzed using BLAST software in GenBank (www.ncbi.nlm.nih.gov). Following the comparison between the sequencing results and the standard sequences in NCBI, 10 species of *Lactobacillus* were detected (Aranishi et al., 2005).

### 2.5 Adhesion of *Lactobacillus* species to S–HA

The adherence ability of the *Lactobacillus* species to human S–HA was determined according to Terai et al. study (2015). The 24‐h culture of 10 isolated *Lactobacillus* species was rinsed 2–3 times with a phosphate‐buffered saline solution (PBS solution) and adjusted to an OD_550_ of 1. Then, 10 mL of human saliva was filtered using a 0.22 μm filter (Merck Millipore). It was incubated at 60°C for 30 min and then centrifuged. The filtered saliva was mixed with the hydroxyapatite powder. Then, 5 mg of S–HA (mixture of HA with saliva) was added to a 2‐mL bacteria suspension. It was incubated at 60°C for 1 h in a shaking incubator. Afterward, 1 mL of the collected supernatant was added and mixed with 0.1 mL of 0.5 M EDTA until the remaining hydroxyapatite particles were dissolved.

### 2.6 Adhesion of isolated *Lactobacillus* species to oral mucosal cells

The adherence ability of the *Lactobacillus* species to oral tissues was determined based on a method proposed by Terai et al. (2015). Oral mucosal cells, the KB/C152 cell line, and the HGF3‐PI 53/C502 cell line, which originated from human epidermoid carcinoma and human gingival fibroblasts, respectively, were obtained from the National Cell Bank of Iran (Pasteur Institute). KB and HGF cells were precultured in Dulbecco's Modified Eagle Medium (D‐MEM; GIBCO) and Roswell Park Memorial Institute (RPMI) 1640 medium (Sigma‐Aldrich), respectively, supplemented with 10% Fetal bovine serum (FBS), 1% Penicillin/Streptomycin, l‐glutamine, and nonessential amino acids (GIBCO). The individual cells were cultured in a growth medium containing carbon dioxide (CO_2_) with 95% humidity for cell proliferation for 72 h. Before culture, gelatin‐coated coverslips were placed at the bottom of each well. Then, 0.5 mL of cell suspension and 1 mL of the medium were poured into each well of a six‐well Chamber Slide (Jet Biofil). It was incubated at 37°C with 5% CO_2_ and 95% humidity for 48–96 h. PBS rinse was carried out three times to remove the nonadhering cells. Isolated oral lactobacilli cultured in the MRS broth for 24 h were centrifuged and rinsed three times using a PBS solution. Suspension of the tested bacteria was added to PBS to adjust the OD_600_ to 0.1. Afterward, 0.5 mL of the prepared suspension was added to the cell culture plate and incubated at 37°C with 5% CO_2_ for 3 h. It was rinsed with PBS three times and then fixed with methanol. After staining with a Gram stain kit, the coverslips were removed and observed under a light microscope (Olympus Corporation). *Escherichia coli* ATCC 25922 was used as the positive control, and the medium without bacterial inoculum was used as the negative control.

The number of bacteria adhered to the oral cells was randomly counted and averaged in six different fields per well. The strains were classified based on the number of attachments: <100 weak, 100–300 medium, 300–500 good, and >500 excellent using the method proposed by Abdel–Daim et al. (2012).

### 2.7 Antibacterial activity of *Lactobacillus* species against oral pathogenic bacteria

The antibacterial spectrum of the cell‐free supernatant (CFS) of *Lactobacillus* species isolated from the oral cavity was studied against two oral pathogens using the radial diffusion assay. The two following bacterial species were chosen as examples of oral pathogens: *Aggregatibacter actinomycetemcomitans (A.a)* Y4 ATCC 43718, a Gram‐negative oral bacterium associated with periodontitis (Damgaard et al., 2021), and *Actinomyces naeslundii (A.n)* ATCC1201, a Gram‐positive bacterium responsible for numerous oral infections, including oral multispecies biofilm development (Mashimo et al., 2016), oral lesions (Suzuki & Delisle, 1984), gingivitis, and periodontitis (Ellen, 1976).

First, the CFS of the lactobacilli cultured in the MRS broth (24 h) and centrifuged at 10,000 rpm was sterilized by a 0.22 µm filter (Merck Millipore). The pH of the bacteria supernatant was adjusted to 7 using 1 N NaOH solution (neutralization). Next, the pathogenic bacteria suspension density was prepared to the half McFarland standard (1.5× 10^8^ CFU/mL) in Brain–Heart Infusion (BHI) broth. Then, it was plated onto BHI agar (in a ratio of 1/100 mL). The agar plates were punched with a diameter of 5 mm, and 100 µL of the lactobacilli supernatants of each species were poured into these punches and incubated at 37°C for 1–2 days. The results of inhibition zones were read after 18–48 h together with a positive control antibiotic (0.05–0.1 mg/mL tetracycline hydrochloride and 0.1 mg/L Chlorhexidine) (Balouiri et al., 2016). *Escherichia coli* ATCC 25922 was used as the negative control.

### 2.8 Statistical analysis

The null hypothesis was that there is no difference between the *Lactobacillus* species in oral isolates (the tongue, subgingival plaque, and saliva) of periodontitis patients compared with periodontally healthy individuals.

The *χ* ^2^ test was utilized to compare the frequency of isolates with *Lactobacillus* species, and the Shapiro–Wilk test was used to check data distribution. The data distribution according to the studied strains was normal in all the studied variables. Data description was presented as mean and standard deviation. One‐way analysis of variance was used to compare the mean adhesion indices, and Tukey's post hoc test was used for pair‐by‐pair comparison. To achieve the antibacterial effect of nongrowth halo in different species, one‐way analysis of variance and Tukey's post hoc test were performed. All statistical analyses were performed using SPSS software (version 25). *p* Values lower than .05 were considered to be statistically significant.

## 3 RESULTS

### 3.1 Isolation and identification of *Lactobacillus* species

The bacterial colonies were isolated from the tongue, saliva, and subgingival plaque samples of 59 patients with periodontitis (177 oral isolates) with a mean age of 48 ± 10.6 years and 59 healthy subjects (177 oral isolates) with a mean age of 37 ± 10.8 years. Among the 59 patients with periodontitis, 6 suffered from stage III and stage IV periodontitis, and 52 suffered from stage II periodontitis according to the current periodontitis classification (Caton et al., 2018).

Out of the 354 collected oral isolates, 134 isolates (75.7%) in the control group and 120 isolates (67.7%) in the case group were positive for *Lactobacillus* strains, which was confirmed by polymerase chain reaction–restriction‐fragment‐length polymorphism. Twelve oral isolates from the case group and 21 isolates from the control group showed no growth for *Lactobacillus* in all saliva, subgingival, and tongue samples (67 isolates were excluded). Moreover, 22 oral isolates in the control group and 45 oral isolates in the case group were positive for vancomycin‐resistant Streptococci and yeasts (67 isolates were excluded). As a result, among the 354 oral isolates collected from both groups, 254 oral isolates were included in further experiments.

As shown in Table 1, there was a 30.5% lower frequency of *Lactobacillus* species detection (*p*  =  .001) in subgingival samples in patients with periodontitis compared with healthy individuals. In contrast, the number of positive isolates in saliva and tongue samples showed no significant difference in the two groups.

**Table 1:** Number and frequency of oral *Lactobacillus* species in different oral samples based on the culture method.

Oral samples	Case group (Periodontitis patients) *N* = 59	Control group (Periodontally healthy patients) *N* = 59	*p* Value
	*N*(%)	*N*(%)	
Saliva	53 (89.8%)	47 (79.6%)	0.124
Tongue	47 (79.6%)	49 (83.1%)	0.810
Subgingival plaque	20 (33.9%)	38 (64.4%)	0.001^a^
Total	120 (100)	134 (100)	

^a^ Significant at *α* = .05.

**Figure 1:**
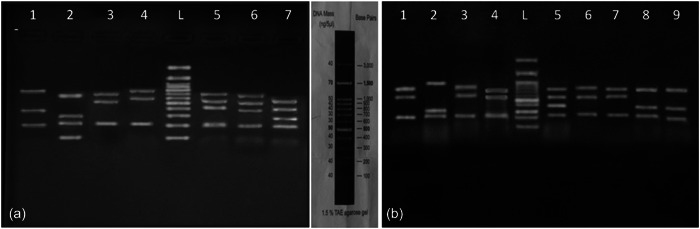
Restriction patterns of 16S rDNA genes resulting from digestion by Taq1 and Hae III restriction enzymes. (a) Taq1 Lanes: 1, *L. acidophilus*; 2, *L. plantarum*; 3, L. casei; 4, *L. paracasei*; 5 and 6, *L. fermentum*; and 7, *L. salivarius*. and (b) Hae III Lanes 1, *L. casei*; 2, *L. crispatus*; 3, *L. gasseri*; 4, *L. vaginalis*; 5, *L. rhamnosus*; 6, *L. paracasei*;7, *L. casei*; and 8 and 9, *L. acidiphilus*. Lane L, ladder 3000 bp.

**Figure 2:**
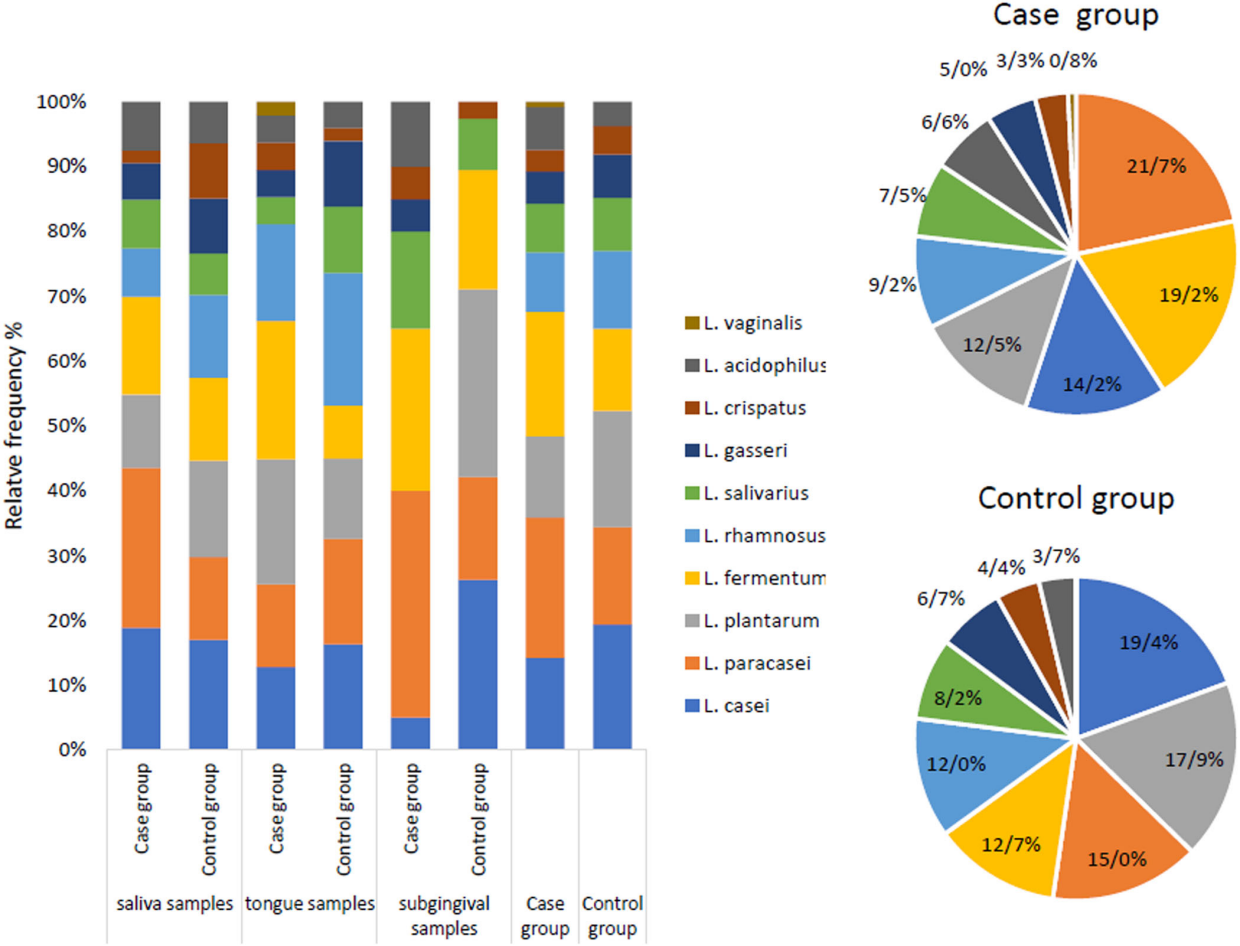
Relative frequency (%) of oral isolates in the case and control samples.

We obtained 10 restriction‐fragment‐length polymorphism patterns from the oral *Lactobacillus* species. In most of the oral samples, we had the same cutting patterns. In some cases, the patterns were repeated. According to Figure 1, out of the 254 positive isolates based on the 16S rDNA sequencing, 10 *Lactobacillus* species were identified in the case group and 9 in the control group. As can be seen in Figure 2, *Lacticaseibacillus paracasei* and *Limosilactobacillus fermentum* were the most frequent species detected in the case group, while *Lacticaseibacillus casei* and *L. plantarum* were the most frequent species in the control group. The least frequent species belonged to *Limosilactobacillus vaginalis* (0.8%) in the case group, which was not detected in the oral samples of the control group. In addition, *L. paracasei*, *L. fermentum*, and *Ligilactobacillus salivarius* were the most abundant strains recovered from subgingival samples of patients with periodontitis, respectively.

### 3.2 Adhesion of *Lactobacillus* species to S–HA and oral mucosal cells

**Figure 3:**
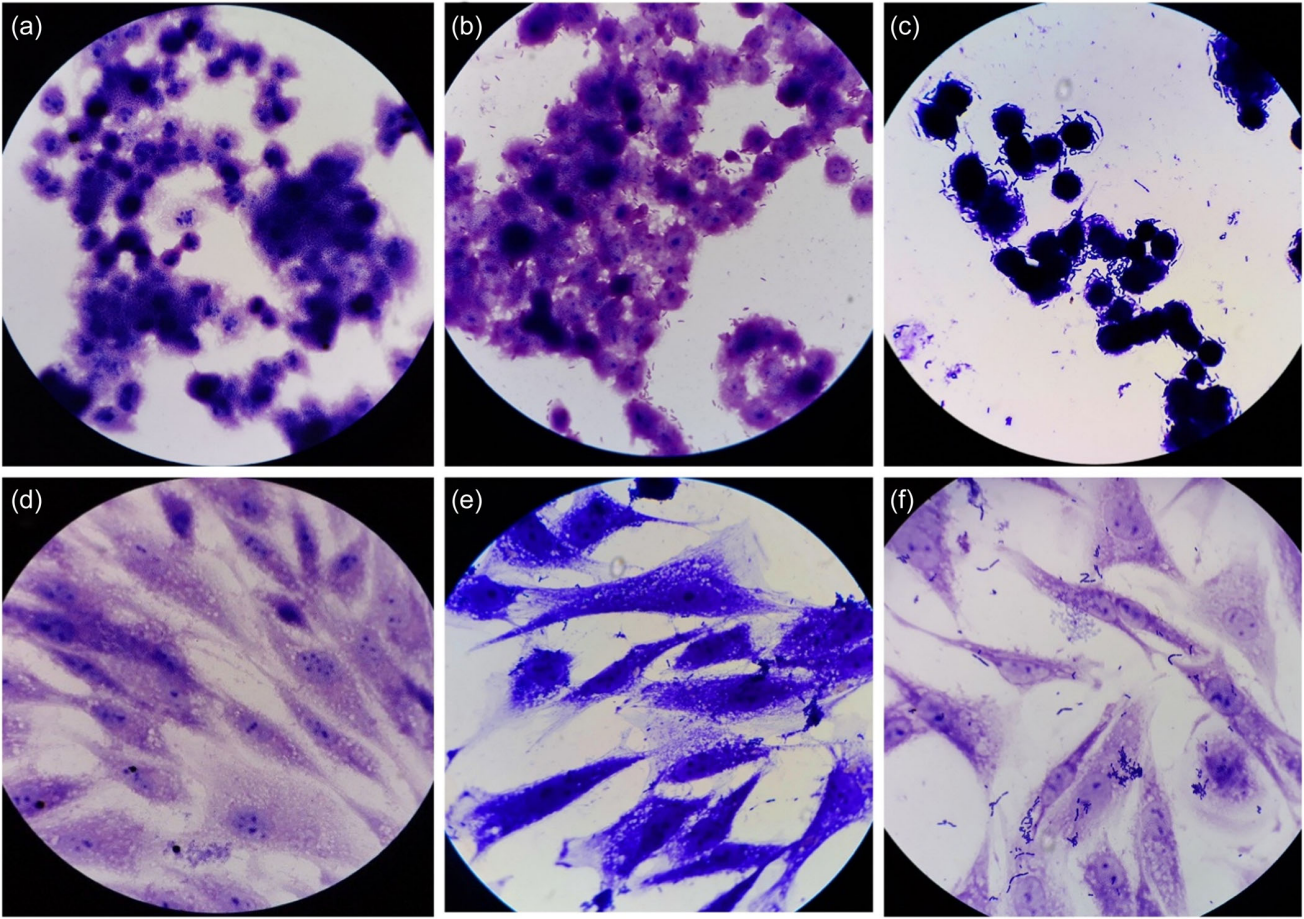
Gram‐stained slides of KB and HGF cells (×100 magnification). (a) KB cells without bacteria (negative control). (b) KB cells adhered to *L. salivarius*. (c) KB cells adhered to *E. Coli* (positive control). (d) HGF cell without bacteria (negative control). (e) HGF cells adhered to *L. salivarius*. (f) HGF cells adhered to *L. fermentum*.

In total, 40 samples out of the 10 species of lactobacilli (four strains were tested from each species) were tested for the adherence ability of *Lactobacillus* species in the case group. Figure 3 shows the *Lactobacillus* adhesion to oral mucosal cells compared with the positive and negative controls.

According to Table 2, *L. salivarius*, *L. fermentum*, and *L. plantarum* showed the highest potency of adhesion to S–HA in the selected strains. Post hoc analysis showed that *L. salivarius* had significantly higher adhesion to S–HA than all other species. In addition, *L. salivarius* had the highest adherence ability to KB cells, with statistically significant differences from others. Moreover, *L. fermentum* and *L. salivarius* had the highest adherence ability to HGF cells. Post hoc analysis showed that *L. salivarius*, *L. fermentum*, *L. acidophilus*, and *L. plantarum* had significantly higher adhesion to HGF cells than other species (Supporting Information: Figures 4, 5, and 6).

**Table 2:** Adherence of oral *Lactobacillus* species to oral mucosal cells (KB and HGF cells) and salivary‐coated hydroxyapatite (S–HA).

Lactobacilli species		Adhesion to H‐SA		Adhesion to KB cells		Adhesion to HGF cells	
		Mean SD	*p* Value	Mean SD	*p* Value	Mean SD	*p* Value
1	*L. casei*	46 ± 13	*p* < .001^a^	71 ± 3	*p* < .001^a^	75 ± 4	*p* < .001^a^
2	*L. paracasei*	48 ± 2		37 ± 4		136 ± 5	
3	*L. plantarum*	59 ± 4		281 ± 13		555 ± 54	
4	*L. fermentum*	60 ± 7.5		320 ± 36		620 ± 73	
5	*L. rhamnosus*	32 ± 7		116 ± 7		61 ± 7	
6	*L. salivarius*	77 ± 6		620 ± 85		610 ± 70	
7	*L. gasseri*	47 ± 2		211 ± 27		95 ± 12	
8	*L. acidophilus*	58 ± 6		301 ± 17		480 ± 27	
9	*L. crispatus*	38 ± 4		300 ± 36		96 ± 21	
10	*L. vaginalis*	46 ± 4		180 ± 18		170 ± 18	

*Note*: Four strains were tested in each *Lactobacillus* species (Total samples: 40).

^a^ Post hoc analysis (Significant at *α* = .05).

### 3.3 Antibacterial activity of isolated *Lactobacillus* species against two oral pathogens

Table 3 compares the inhibition zones of 10 selected lactobacilli species in the case group supernatants (40 samples in total) against *A.a* and *A.n*. The antibiogram results of *A.n* were read after 24 h, and *A.a* results were read after 48 h due to the slower growth rate. Most strains had almost similar antibacterial activity and inhibition zones. However, *Lactobacillus crispatus* and *Lactobacillus gasseri* showed a larger inhibition zone against *A.a, and Lacticaseibacillus rhamnosus and L. acidophilus* showed the highest antibacterial activity against *A.n*. Post hoc analysis confirmed these significant differences (*p* < .001).

**Table 3:** Mean inhibition zones of the selected oral *Lactobacillus* strains against oral pathogens.

Lactobacilli species		Inhibition clear zone *A.a* a		Inhibition clear zone *A.n* a	
		Mean SD	*p* Value	Mean SD	*p* Value
1	*L. casei*	6 ± 0.8	*p* < .001^b^	5 ± 0.2	*p* < .001^b^
2	*L. paracasei*	8 ± 0.2		7 ± 0.4	
3	*L. plantarum*	7 ± 0.2		7 ± 0.2	
4	*L. fermentum*	8 ± 0.2		6 ± 0.3	
5	*L. rhamnosus*	5 ± 0.1		8 ± 0.1	
6	*L. salivarius*	6 ± 0.1		6 ± 0.1	
7	*L. gasseri*	9 ± 0.1		6 ± 0.2	
8	*L. acidophilus*	7 ± 0.1		8 ± 0.0	
9	*L. crispatus*	10 ± 0.2		6 ± 0.7	
10	*L. vaginalis*	8 ± 0.0		5 ± 0.0	

*Note*: Four strains were tested in each *Lactobacillus* species (Total samples: 40).

^a^ Oral pathogens: (*A.a: Aggregatibacter actinomycetemcomitans*, *A.n: Actinomyces naeslundii*).

^b^ Post hoc analysis (Significant at *α* = .05).

## 4 DISCUSSION

The traditional approaches to control dental plaque‐related diseases were based on nonspecific mechanical removal of all the beneficial and nonbeneficial plaques (Johnston et al., 2021). However, modern treatment approaches have recently emphasized the inhibition of specific small groups of organisms, single species, or even the main pathogenic agents (Allaker & Stephen, 2017). Furthermore, the increase in antibiotic resistance resulted in the search for alternative products or treatment strategies (Myneni et al., 2020). Several species of Lactobacilli, known as probiotics, have been used recently to treat periodontitis (Kuru et al., 2017; Laleman et al., 2020; Pelekos et al., 2020; Schlagenhauf et al., 2020; Shimauchi et al., 2008; Silva et al., 2022; Teughels et al., 2013). It seems necessary to conduct numerous experiments to identify and test their properties to optimize the results of these specific treatments. This was the first study conducted on oral samples of an Iranian population, comparing periodontitis patients and periodontally healthy individuals.

This research used unique growth and molecular methods, 16S rDNA genes polymerase chain reaction–restriction‐fragment‐length polymorphism, and universal primers to precisely detect the lactobacilli species. The m‐MRS medium facilitated the detection of *Lactobacillus* species and hindered the growth of other LAB. In addition, the 16S rDNA genes polymerase chain reaction–restriction‐fragment‐length polymorphism and sequencing considerably facilitated bacterial strain identification.

This study revealed that the individuals with periodontitis have a lower relative frequency of oral *Lactobacillus* species compared with the control group, especially in the subgingival samples, which can be due to the establishment and function of pathogenic bacteria in the periodontal tissues.

Furthermore, different *Lactobacillus* species were identified from the oral isolates of the case group and the control group. Among them, *L. casei* and *L. plantarum* were the dominant species in the control group, and *L. paracasei* and *L. fermentum* were the most frequent species in the case group. Most of the identified species in the two groups were consistent with previously reported findings (Gupta, 2011; Koll‐Klais et al., 2005). However, there were differences in the frequency of the species in each group. In the study by Ahrne et al., the most frequent species in the samples of the healthy participants were *L. plantarum* and *L. rhamnosus* (Ahrné et al., 1998). Colloca et al. found that *L. fermentum*, *L. plantarum*, *L. salivarius*, and *L. rhamnosus* were the most frequent species in the oral cavity of healthy participants (Colloca et al., 2000). As reported by Koll–Klais et al., the most prevalent strains in the healthy participants were *L. gasseri* and *L. fermentum*, and the most frequent strain in the case group was *L. plantarum* (Koll–Klais et al., 2005). The similarity and differences of species found in oral isolates can be due to patients' various food and dietary habits (Sornplang & Piyadeatsoontorn, 2016).

According to previous reports, the ability to adhere to mucosal host surfaces has always been an essential property among bacterial strains used as probiotics (FAO/WHO, 2002). This study showed that *L. salivarius* and *L. plantarum* had the highest adherence activity to KB, HGF cells, and S–HA. Thus, probiotic bacteria such as *L. plantarum* and *L. salivarius* can directly adhere to the oral mucosal cells, develop oral biofilms in the saliva, reside on the tongue surface, and exert healthy effects. According to Bosch et al. (Colloca et al., 2000), 10 and 38 isolates from the salivary strain of healthy children showed higher adherence potential than the commercial species of *Streptococcus salivarius* K12 and *Limosilactobacillus reuteri*, respectively. This proves that probiotics isolated from the oral cavity had a higher capacity to develop biofilms and inhibit the growth of pathogens than commercial probiotic products. This emphasizes the importance of identifying and detecting the probiotic properties of healthy individuals' oral cavity to optimize probiotic treatments. In a study by Terai et al., the adherence ability of *Lactobacillus* species was evaluated by adhesion to S–HA and oral epithelial cells derived from human buccal mucosa carcinoma and human tongue carcinoma. Only *L. fermentum*, *L. gasseri*, and *L. casei* showed adhesion to S–HA, and *L. crispatus* had higher adherence activity to human tongue carcinoma cells (Terai et al., 2015).

In addition to preserving the balance of oral microbiota, probiotics improve oral and periodontal health by producing antibacterial metabolites. In this study, most oral *Lactobacillus* supernatant showed antibacterial activity against *A.n* and *A.a* after neutralization. This proves that organic acids are not the only antimicrobial substances in the supernatant of bacteria, and *Lactobacillus* might potentially produce bacteriocins or other antibacterial substances in the supernatant. Moreover, *L. crispatus* and *L. gasseri* showed higher antibacterial activity against these two pathogens. These findings are in agreement with the study carried out by Terai et al., in which they pointed out that the supernatants of most oral LAB showed antibacterial activity against *P. gingivalis*. Furthermore, *L. crispatus* showed antibacterial activity against *A.a* after neutralization (Terai et al., 2015).

Studies investigating the antimicrobial activity of various species of LAB were conducted against different pathogens using different methods (Ben Taheur et al., 2016; Koll‐Klais et al., 2005; Samot & Badet, 2013). These differences might affect the antibacterial test results and complicate comparison. However, there were similarities among the species with the most antibacterial activities. Azizian et al. revealed that *L. gasseri*, *L. salivarius*, *L. crispatus*, and *Lactobacillus curvatus* could inhibit the growth of pathogenic bacteria (Azizian et al., 2021). Hirasawa et al. demonstrated that *L. casei*, *L. fermentum*, and *L. gasseri* showed intense antibacterial activity against *P. gingivalis* (Hirasawa & Kurita–Ochia, 2020). Rahne et al. found that *L. paracasei* manifested the highest antimicrobial activity against *streptococcus mutans*, followed by *L. fermentum* and *L. casei/rhamnosus* (Rahne et al., 2021). Sookhee et al. isolated two LAB species (*L. paracasei* and *L. rhamnosus*) from healthy participants' oral cavities, demonstrating antimicrobial activity against *S. mutans*, *Streptococcus sanguinis*, *S. salivarius*, *Staphylococcus aureus*, *Actinomyces viscosus*, *P. gingivalis*, and *Candida* (Sookkhee et al., 2001). Koll–Klais et al. reported that *L*. paracasei, *L. plantarum*, *L. rhamnosus*, and *L. salivarius* showed the highest antimicrobial activity (Koll–Klais et al., 2005). However, Testa et al. found no antagonistic effect between oral LAB (*L. casei*, *L. rhamnosus*, *L. plantarum*, and *L. salivarius*) and anaerobes *Fusobacterium nucleatum* and *Prevotella intermedia* (Testa et al., 2003).

The present study contributes to knowledge about oral lactobacilli in the Iranian population, which may be beneficial in future randomized clinical trials and drug discovery. The main limitation of this study was its case selection and finding the matched patients in the case and control groups according to the inclusion and exclusion criteria. Moreover, the authors intended to work on *P. gingivalis* as the keystone bacterium in periodontal disease development, but due to its lack of availability, *A.a* and *A.n* were selected as proper substitutes for oral pathogens.

The selection of *Lactobacillus* species as probiotics was based on their antibiotic activity against bacterial pathogens and their ability to adhere to epithelial cells (Zhang et al., 2020). Based on our results, consistent with previous findings (Hirasawa & Kurita–Ochia, 2020; Samot & Badet, 2013), *L. crispatus* and *L. gasseri*, *L. salivarius*, and *L. fermentum* have excellent probiotic potential. More strain‐based studies must be carried out to investigate and detect the metabolites and antibacterial substances produced by the selected species to determine the safe usage of each *Lactobacillus* in terms of its unique characteristics for specific purposes.

## 5 CONCLUSION

The present study aimed to assess the properties in terms of the probiotic potential of oral *Lactobacillus* species for the probiotic treatment of periodontal diseases. In summary, it can be concluded that *L. crispatus* and *L. gasseri* strains, on account of their higher antibacterial properties, and *L. salivarius* and *L. fermentum*, due to their high adherence ability, might be appropriate options for the probiotic treatment of periodontal diseases. In addition, the lower frequency of *Lactobacillus* strain detection in the subgingival plaque of patients with periodontitis indicates the presence and function of pathogenic bacteria in periodontal tissue and the imbalance of oral microbial flora. Further studies should be conducted to assess the safety of probiotic interventions using these strains in patients with periodontal disease.

## AUTHOR CONTRIBUTIONS

Arghavan Etebarian and Tahere Sheshpari contributed to the conception and design of the study. Tahere Sheshpari designed and carried out the experiments. Abouzar Moradi and Tahere Sheshpari contributed to the data acquisition. Kourosh Kabir performed the statistical analysis. Arghavan Etebarian, Hanieh Sadeghi, and Avin Hafedi wrote the first manuscript draft. All authors contributed to the article revision and approved the submitted version.

## CONFLICT OF INTEREST STATEMENT

The authors declared no potential conflicts of interest concerning the research, authorship, and publication.

## Supporting information

Supporting information.

Supporting information.

Supporting information.

## Data Availability

The data that support the findings of this study are available on request from the corresponding author. The data are not publicly available due to privacy or ethical restrictions.
