# Supplementary figures and images for: Oral Lactobacillus species and their probiotic capabilities in patients with periodontitis and periodontally healthy individuals
Source: Clin Exp Dent Res. 2023 Apr 20;9(5):746–56. doi: 10.1002/cre2.740 (PMC10582226; doi:10.1002/cre2.740)

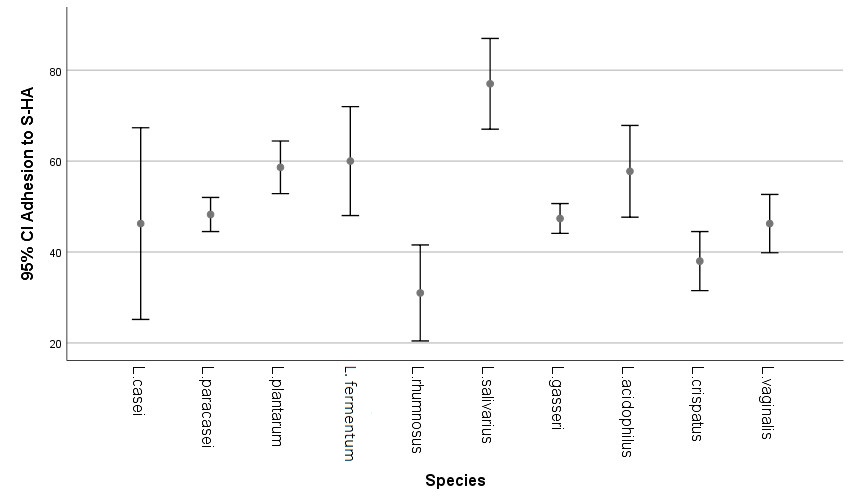


**Supplementary Figure 4.** *Lactobacillus* species adhesion to S-HA

Supplement: Supplementary file 1 — Supporting information. [file CRE2-9-746-s001.docx]

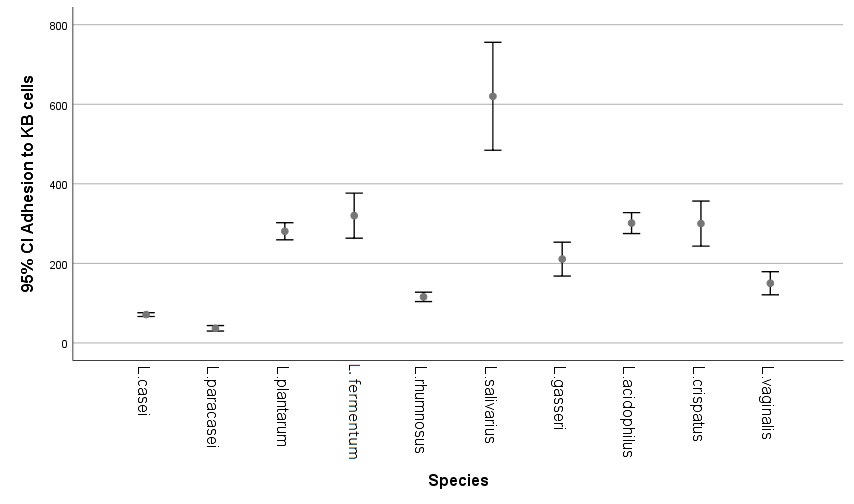


**Supplementary Figure 5.** *Lactobacillus* species adhesion to KB cells

Supplement: Supplementary file 2 — Supporting information. [file CRE2-9-746-s003.docx]

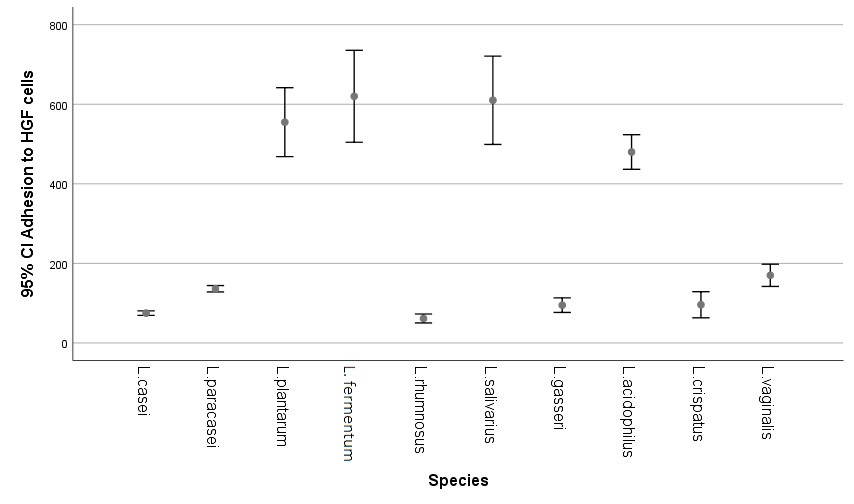


**Supplementary Figure 6.** *Lactobacillus* species adhesion to HGF cells

Supplement: Supplementary file 3 — Supporting information. [file CRE2-9-746-s002.docx]
